# Comprehensive Transcriptome Study to Develop Molecular Resources of the Copepod *Calanus sinicus* for Their Potential Ecological Applications

**DOI:** 10.1155/2014/493825

**Published:** 2014-05-20

**Authors:** Qing Yang, Fanyue Sun, Zhi Yang, Hongjun Li

**Affiliations:** ^1^Key Laboratory for Ecological Environment in Coastal Areas (SOA), National Marine Environmental Monitoring Center, Dalian 116023, China; ^2^Department of Reconstructive Sciences, Center for Regenerative Medicine and Developmental Biology, University of Connecticut Health Center, Farmington, CT 06030, USA; ^3^Harbin University of Science and Technology, Rongcheng Campus, Rongcheng 264300, China

## Abstract

*Calanus sinicus* Brodsky (Copepoda, Crustacea) is a dominant zooplanktonic species widely distributed in the margin seas of the Northwest Pacific Ocean. In this study, we utilized an RNA-Seq-based approach to develop molecular resources for *C. sinicus*. Adult samples were sequenced using the Illumina HiSeq 2000 platform. The sequencing data generated 69,751 contigs from 58.9 million filtered reads. The assembled contigs had an average length of 928.8 bp. Gene annotation allowed the identification of 43,417 unigene hits against the NCBI database. Gene ontology (GO) and KEGG pathway mapping analysis revealed various functional genes related to diverse biological functions and processes. Transcripts potentially involved in stress response and lipid metabolism were identified among these genes. Furthermore, 4,871 microsatellites and 110,137 single nucleotide polymorphisms (SNPs) were identified in the *C. sinicus* transcriptome sequences. SNP validation by the melting temperature (*T*_*m*_)-shift method suggested that 16 primer pairs amplified target products and showed biallelic polymorphism among 30 individuals. The present work demonstrates the power of Illumina-based RNA-Seq for the rapid development of molecular resources in nonmodel species. The validated SNP set from our study is currently being utilized in an ongoing ecological analysis to support a future study of *C. sinicus* population genetics.

## 1. Introduction

Copepods are an extremely ancient and diverse arthropod group. They have evolved into more species than any other multicellular animal group [1, 2]. To date, more than 12,000 copepod species have been recognized all over the world [1] and over 700 species are identified in the China Seas. They pervade various aquatic habitats and show local adaptation to rapid environmental changes. Copepod adults are very small in size, which is mainly limited to 1–4 mm. However, they show great diversity in their morphology, physiology, and life strategies, which makes them very suitable for studying a variety of fundamental biological processes. Although copepods are critical species for the world's aquatic ecosystems, the available genomic resources are still limited, and sequencing efforts have been carried out for a small number of well-studied species [2]. Of these, the parasitic copepods sea lice have received great attention because of their adverse effect on the global salmon aquaculture industry [3]. The developed expressed sequence tags (ESTs) enabled studies to investigate host-parasite interactions at the molecular level and provided promising targets for vaccine development [4]. Moreover, a large number of ESTs are available for* Calanus finmarchicus* [5–7], a key planktonic species from the North Atlantic Ocean. This genomic information supported the development of a cDNA microarray, which was utilized to investigate the physiological responses to environmental variations [8]. Several functional genes with important physiological roles were identified by mining EST and 454 pyrosequencing data in* C. finmarchicus* [9–11]. Just recently, Ning et al. [12] performed the first large-scale transcriptome sequencing for* Calanus sinicus* to identify putative transcripts involved in growth, lipid metabolism, molting, and diapause process. Although more than 50,000 high-quality ESTs were obtained, more transcriptome data are needed to present a full view of this transcriptome organization and provide complete gene sets to facilitate future genomic and genetic studies in this species.

*Calanus sinicus* Brodsky is a planktonic copepod widely distributed in the shelf ecosystem of East Asia [13] and it dominates the mesozooplankton in the shelf water of Bohai Sea, Yellow Sea, East China Sea, and Inland Sea of Japan. Its adults, larvae, and eggs are the main food source for many commercially important fish, such as sardine and anchovy. Therefore, it is recognized as a key secondary producer that links phytoplankton and higher trophic level organisms and its population dynamics greatly impact the entire marine ecosystem. The spatial distribution of* C. sinicus* has changed in the continental shelf waters of China Seas as a result of global climate change [14, 15], raising concerns about future climate-driven shifts in the geographical distribution of* C. sinicus*. These shifts in the biogeography of* C. sinicus *call for a better understanding of organism-environment interactions. However, the way that this organism responds physiologically to environmental variations is not well known, as well as the adaptive capacity of this species to elevated temperature and ocean acidification induced by climate change.

With the rapidly declining cost of next generation sequencing, RNA sequencing (RNA-Seq) approaches have been more widely applied to population genetic and molecular ecology studies of nonmodel species [16, 17]. In this study, we describe the utilization of RNA-Seq to capture a significant portion of the* C. sinicus *transcriptome (expressed portion of the genome), stress-related expression signatures, and thousands of potential molecular marker loci in an Illumina HiSeq next generation sequencing run. Our results significantly deepen the pool of molecular resources available for this taxon and serve as a guide for similar studies in related copepod taxa.

## 2. Materials and Methods

### 2.1. *Calanus sinicus* Sample Collection

*Calanus sinicus *samples for transcriptome sequencing were collected from the Yellow Sea (38°45′N, 121°45′E) with a 500 *μ*m mesh zooplankton net in May 2013. Zooplankton were preserved in fresh seawater temporarily and transported to the Zooplankton Ecology Lab of National Marine Environmental Monitoring Center (Dalian, China).* C. sinicus* were manually picked up with the aid of a stereomicroscope and preserved at −80°C pending RNA extraction.

### 2.2. RNA Extraction, Library Construction, and Illumina Sequencing

Total RNA was extracted from a pool of about 50 individuals using RNeasy Mini Kit (Qiagen, Germany) following the manufacturer's instruction. The concentration of total RNA was determined by NanoDrop (Thermo Scientific, USA) and the RNA integrity value was checked with a RNA 6000 Pico LabChip on an Agilent 2100 Bioanalyzer (Agilent, USA). High-quality RNA was then provided for RNA-Seq library construction and Illumina sequencing. A cDNA library was constructed with ~5 *μ*g initial DNase-treated total RNA following the protocols of the TruSeq RNA sample preparation kit (Illumina). After poly(A) mRNA was enriched by beads with Oligo (dT), a fragmentation buffer was added for shearing mRNA to short fragments (200–700 bp). Taking these short fragments as templates, a random hexamer-primer was used to synthesize the first-strand cDNA, and then the second-strand was amplified. The double-stranded cDNA was purified with the Qiagen PCR extraction kit, and the short fragments were connected with sequencing adaptors. The library was PCR amplified and the final library had the yields of ~30 *μ*L of 19.8–21.4 ng/*μ*L with an average length of ~270 bp. After KAPA quantitation and dilution, the library was sequenced on an Illumina HiSeq 2000 instrument with 100 bp paired-end (PE) read chemistry by Majorbio Biotechnology Corporation (Shanghai, China).

### 2.3. *De Novo* Assembly and Transcriptome Analysis

**Figure 1:**
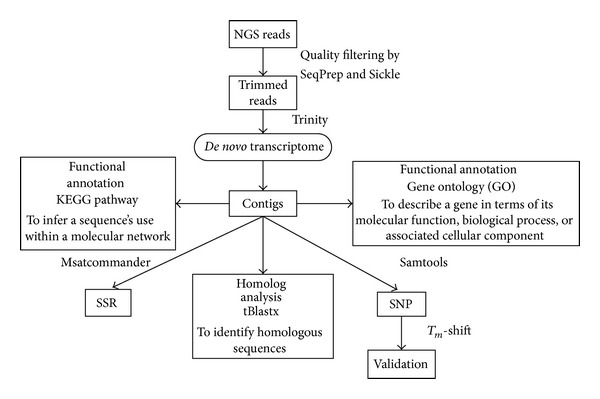
Schematic presentation of the copepod* Calanus sinicus *transcriptome analysis. After sequencing, raw reads were trimmed by stripping the adaptor sequences and ambiguous nucleotides using SeqPrep and Sickle.* De novo* assembly was performed using Trinity. The assembled contigs were used for three separate analyses: (a) gene identification and annotation analysis; (b) pathway analysis; and (c) SSR and SNP screening and validation.

Transcriptome raw sequences were subjected to a series of assembly and annotation programs (Figure 1).* De novo* assembly of short reads originating from Illumina sequencing was performed using Trinity [18]. Before assembly, raw reads were trimmed by stripping the adaptor sequences and ambiguous nucleotides using SeqPrep (https://github.com/jstjohn/SeqPrep) and Sickle (https://github.com/najoshi/sickle). Reads with quality scores less than 20 and lengths below 20 bp were removed. The resulting cleaned and filtered high-quality sequences were used in the subsequent assembly with the default settings including a fixed* k*-mer size of 25 as suggested.

### 2.4. Gene Annotation, Ontology, and Pathway Analysis

The resulting transcripts from Trinity assembly were used as queries for ORF prediction. A set of utilities included in the Trinity software were employed to extract the likely coding regions from Trinity transcripts. Gene annotation was then performed on the protein sequences with predicted ORF and the nucleotide sequences without predicted ORF, respectively. Sequence homology searches of the protein sequences with predicted ORF were performed using BLASTP program against sequences in NCBI nonredundant (nr) protein database, STRING database (http://string-db.org/), and KEGG GENES database (http://www.genome.jp/kegg/genes.html), while the contigs without predicted ORF were used as queries for BLASTX searches. The cutoff Expect value (*E*-value) was set at 1*e* − 5 and only the top hit result against known sequences was assigned as the annotation. Gene ontology (GO) analysis for biological process, molecular process, and cellular component was processed with Blast2GO 2.5.0 [19], which is an automated tool for the assignment of gene ontology terms. The final annotation file was generated after gene-ID mapping, GO term assignment, annotation augmentation, and generic GO-slim processes. All the annotated contigs were categorized with regard to biological process, cellular component, and molecular function at level 2. They were used to determine the GO term, COG term, and further KEGG pathway analysis.

### 2.5. Identification of Microsatellites and Single Nucleotide Polymorphisms (SNPs)

The microsatellite mining was performed using the program Msatcommander [20]. The search criteria were set based on the number of repeat motifs: 7 for dinucleotides, 5 for trinucleotides, tetranucleotides, and pentanucleotides, and 4 for hexanucleotides. We implemented the SNP discovery process using Samtools (http://samtools.sourceforge.net/). Briefly, the Trinity-assembled transcripts were used as reference sequences. SNPs were determined as superimposed nucleotide peaks where two or more reads contained polymorphisms at the variant allele with the default parameter. With the aim of avoiding false positive SNPs due to sequencing errors (which may therefore be monomorphic loci), only both variants with a minimum variant count of 2 high-quality (HQ) bases and a minimum site depth of 8 (HQ bases) were considered as putative SNPs.

### 2.6. Validation of SNP Markers with Melting Temperature- (*T*_*m*_-) Shift Method

The *T*_*m*_-shift method [21] was used to genotype SNPs. For each SNP locus, the primer set included one common reverse primer (CR) and two forward allele-specific primers (AS1 and AS2), with the 3′ terminal base of each specific primer matching one of the SNP alleles. The common primer was typically placed no more than 20 bp downstream from the SNP for favoring allele discrimination. GC tails of different lengths, 14 bases for one primer and 6 for the second, were added to each of the two allele-specific primers to discriminate melting curve of amplification products. As a rule, the long tail was attached to the allele-specific primer with the higher *T*_*m*_ base (G or C) at its 3′ end, and the short tail was attached to the other allele-specific primer with lower *T*_*m*_ base (A or T).

For SNP polymorphism analysis, 30 wild individuals of* C. sinicus* were collected from the Northern Yellow Sea. Total DNA was extracted from each single individual using genomic DNA isolation kit (Foregene, China). Allele-specific PCR was carried out in a final volume of 25 *μ*L containing 10 ng DNA, 1 × PCR SYBR Premix Ex* Taq* buffer (Takara), and 0.2 *μ*M each of the 3 primers. The PCR program was as follows: initial denaturation at 95°C for 30 s, followed by 40 cycles of 3-step amplification profile of 5 s at 95°C for denaturation, 30 s at 60°C for annealing, and 20 s at 72°C for extension. Melting curves were obtained using ABI 7500 real-time thermal cycler with the default “dissociation step” to measure the fluorescence intensity of the PCR product in a linear denaturation ramp from 65°C to 95°C. POPGENE 32 [22] was used to calculate observed and expected heterozygosities (*H*_*o*_ and *H*_*e*_).

## 3. Results

### 3.1. Sequencing of Short Expressed Reads from* Calanus sinicus* Transcriptome

Illumina-based RNA-Seq was conducted, generating a total of 58.9 million 100 bp paired-end (PE) reads. After trimming of low-quality reads (quality scores <20) and short read sequences (less than 20 bp), a total of 57.7 million high-quality sequences (98.0%) were obtained (Table 1). These sequences were selected for further analysis. All of the sequences with raw read data were deposited at the NCBI sequence read archive (SRA) database (SRP032493).

### 3.2. *De Novo* Assembly

**Figure 2:**
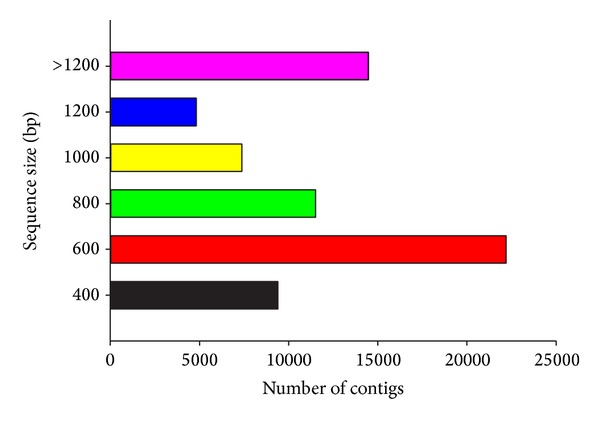
Size distribution of the assembled contigs in the* Calanus sinicus *transcriptome.

Assembly of the 57.7 million cleaned short reads using Trinity resulted in approximately 69,751 contigs with N50 of 1,127 bp (Table 1). These 69,751 contigs with an average length of 928.8 bp were designated as the final transcriptome of* C. sinicus*. Among the 69,751 contigs, 31,581 (45.3%) were less than 600 bp, while 23,695 (34.0%) ranged from 600 to 1,200 bp, whereas 14,485 (20.8%) were more than 1,200 bp in length (Figure 2).

### 3.3. Gene Annotation

**Figure 3:**
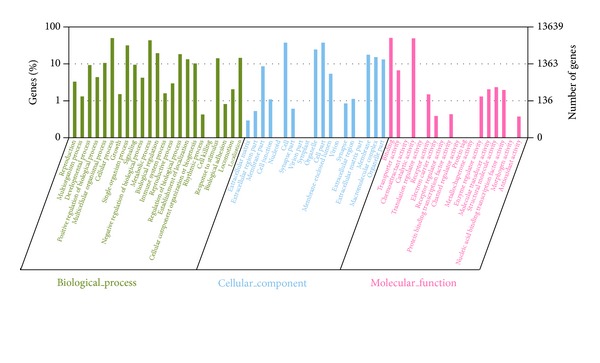
Gene ontology classification of assembled unigenes of* Calanus sinicus* transcriptome on biological process, cellular component, and molecular function levels.

All the assembled Trinity contigs were used as queries in BLASTP and BLASTX searches. A total of 58,885 assembled contigs had significant (*E*-value ≤1*e* − 5) hits against the nr protein database, representing 43,417 unique proteins. After the initial BLAST searches, BLAST2GO analysis was conducted to categorize the known genes into the level 2 functional groups (Figure 3). A total of 60 GO terms were assigned to 13,639 unigenes, including 23 (38.3%) biological process terms, 19 (31.7%) cellular component terms, and 18 (30.0%) molecular function terms. For biological process, the genes involved in the cellular and metabolic processes were highly represented. For the cellular component, the cell was the most represented GO term, followed by the cell part. Molecular function mainly included binding and catalytic activity. GO annotation identified 1,934 unigenes involved in response to stimulus (GO: 0050896) (Table 2; see Table S1 in Supplementary Material available online at http://dx.doi.org/10.1155/2014/493825). This category may be of interest to ecotoxicology researchers, since the responses to environmental stress can be used as biomarkers to evaluate the biological effects of different types of pollutants in aquatic animals.

KEGG pathway analysis was also carried out in addition to GO analysis for the annotated unigenes, which is an alternative approach to categorizing gene functions with an emphasis on biochemical pathways. Enzyme commission (EC) numbers were assigned to 14,553 unigenes involved in 324 different pathways. Summary of the sequences of these pathways is shown in Table 3. Among the 14,533 genes with KEGG annotation, 45.5% were classified into the genetic information processing (GIP) group with most of them involved in replication and repair, folding, sorting and degradation, transcription, and translation. Sequences classified into the metabolism accounted for 42.8% of the KEGG annotated sequences. The well-represented metabolic pathways were enzyme families, carbohydrate metabolism, amino acid metabolism, and energy metabolism. Cellular processes were represented by 18.3% of the KEGG annotated sequences. Cell motility, cell growth and death, immune system, and endocrine system were well represented. Furthermore, 15.2% of the sequences were classified into environmental information processing (EIP) including signal transduction, signaling and interaction molecules, and membrane transport. Lipids play an important role in the lifecycle of the copepod; therefore, the genes involved in lipid metabolism were also identified (Table 2, Table S1).

**Figure 4:**
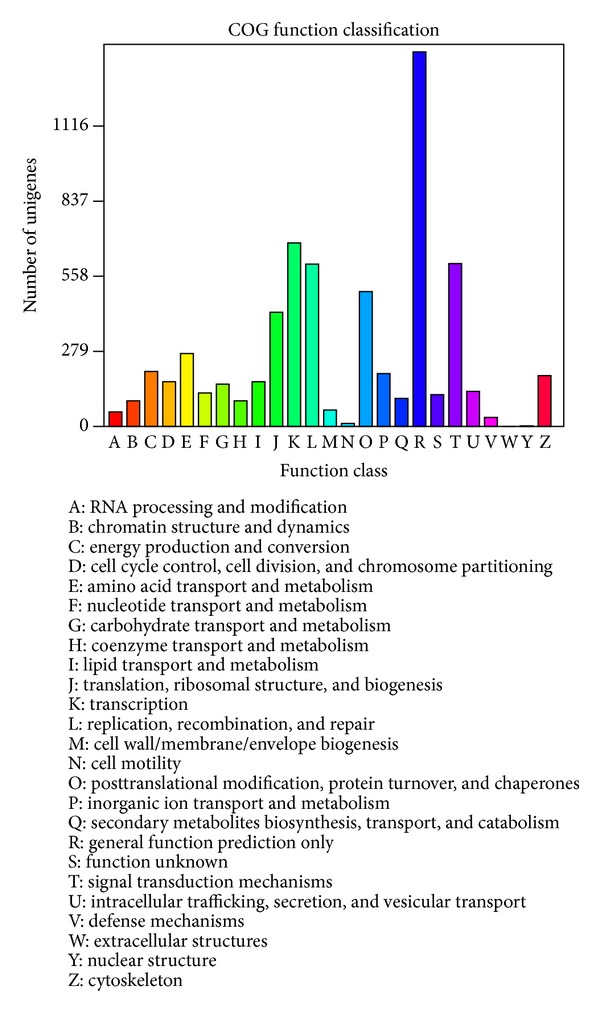
Clusters of orthologous groups (COG) classification. In total, 6,383 of the 43,417 sequences with nonredundant (nr) protein hits were grouped into 25 COG classifications.

Similarly, COG-annotated putative proteins were classified functionally into at least 25 molecular families, such as the cellular structure, biochemistry metabolism, molecular processing, signal transduction, gene expression, and immune defense. All of these families correspond to the categories observed in GO analysis (Figure 4).

### 3.4. Microsatellite and SNP Identification

A total of 4,871 microsatellites were identified. Most microsatellites were trinucleotide (92.4%) and dinucleotide (4.8%) repeats (Table 4). AGG was the predominant trinucleotide repeat motif among these repeats and showed a frequency of 20.7%.

**Figure 5:**
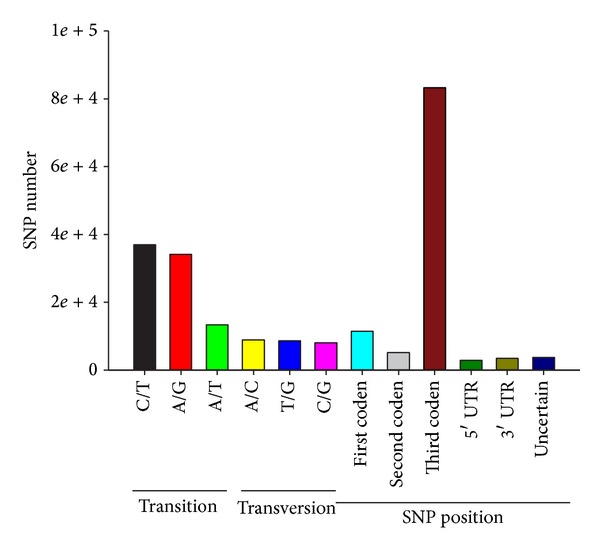
Classification of single nucleotide polymorphisms (SNPs) identified in the* Calanus sinicus* transcriptome.

Alignment of sequence data to the Trinity reference transcriptome revealed the presence of 110,137 putative SNPs, including 71,213 transitions (C/T: 37,017; A/G: 34,196) and 38,924 transversions (A/T: 13,346; A/C: 8,904; T/G: 8,619; C/G: 8,055) (Figure 5), with C/T (33.6%) being the most common and C/G (7.3%) being the least common. The frequency of SNPs in the transcriptome was 3.01 per 1 kb. Most (83,270, 75.6%) SNPs occurred at the third position in a codon and were often referred to as synonymous SNPs, which do not alter the translated amino acid residue.

**Figure 6:**
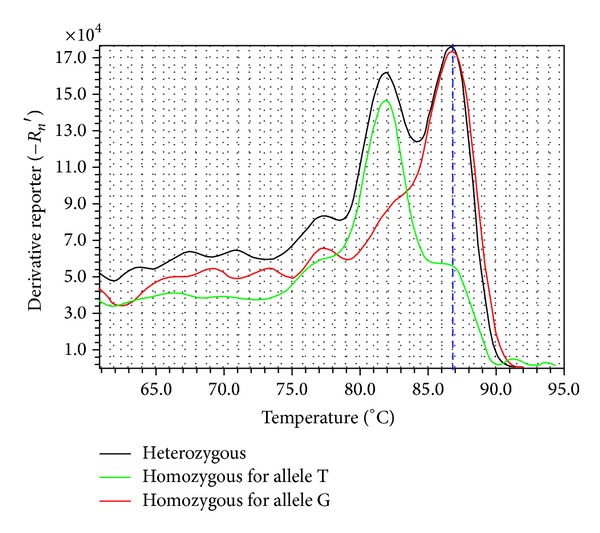
Melting curve of locus CsSNP02 genotyped with *T*_*m*_-shift method. GC tails of different lengths were added to allele-specific primers. Samples homozygous for allele A or T will be amplified with the short GC-tailed primer and show lower temperature peak. Samples homozygous for allele G or C will be amplified with the long GC-tailed primer and show higher temperature peak. Samples heterozygous will show both temperature peaks.

According to the frequency of mutation and the conservation of flanking sequences, 51 putative SNPs were selected for validation with *T*_*m*_-shift primers. Of the 51 primer pairs, 6 (11.8%) did not amplify any product and 29 (56.9%) failed because of amplification failure of one allele-specific primer. Sixteen (31.4%) were successful and showed biallelic polymorphisms among 30 individuals (Table 5). In Figure 6, we show an example of *T*_*m*_-shift genotyping assay for the locus CsSNP02 based on allele-specific PCR.

## 4. Discussion

Genomic tools play an important role in advancing our knowledge of biology at all levels, from genes to ecosystems. Generally, ecological studies often focus on nonmodel species, which lack genomic information. The development of next-generation sequencing techniques provides an exciting opportunity to explore the physiological ecology of organisms of interest. The pelagic copepod* C. sinicus* is a key zooplankton species in the shelf ecosystem of Northwest Pacific Ocean. Previous biological and ecological studies suggested a considerable diversity of physiological responses of* C. sinicus* to different environment conditions as well as distributional variations associated with monsoon, coastal currents, and temperature [23–25]. There are significant gaps in our understanding of the physiological mechanisms driving this diversity thus far. In ecological studies, a transcription-level assessment of physiological state can contribute important information about individuals in a population. Molecular methods allow researchers to identify gene expression levels involved in any physiological responses and measure sublethal effects on the gene level.

A wide array of biochemical, cellular, and whole-organism markers have been applied to evaluate the biological effects of different types of pollutants in aquatic animals and assess the status of marine ecosystems. The identification of several stress and immune-related genes is of great interest to ecologists due to their potential as biomarkers for environmental contamination. In the* C. sinicus *transcriptome developed in this study, we identified several types of heat shock protein (HSP) genes, including HSP90, HSP70, HSP60, HSP40, and HSP10. HSP family plays an important role in thermal tolerance, which is necessary for protein folding and regulation of the heat shock response [26, 27]. The characterization of these genes provides an opportunity to understand the molecular signals involved in the thermal tolerance of planktonic copepods and will help understand the effects of global climate change on marine species with an extensive geographical distribution range. Another important gene family identified in this study was cytochrome P450 (CYP). CYPs are one of the major phase I-type classes of detoxification enzymes, which can catalyze the oxidation of a wide variety of exogenous compounds or xenobiotics [28]. We also identified a large number of glutathione S-transferases (GSTs), which are a superfamily of multifunctional phase II enzymes primarily involved in the detoxification of endogenous electrophiles. Superoxide dismutases (SODs) are an ubiquitous family of enzymes that function to efficiently catalyze the dismutation of superoxide anions [29]. Cu/Zn SOD and Mn-SOD have been characterized in the copepod* Tigriopus japonicus*, and expression level could be inducible by heavy metals and B[*α*]P, which indicated their potential as biomarkers for the risk assessment of these environmental pollutants [30]. Further studies in this direction can help understand the changes in the expression of CYPs, GSTs, and SODs under toxic stressors and explore the relation between gene expression and oxidative activity.

True diapause is an important life strategy shared by many copepods. To survive long periods of low food availability, copepods undergo an ontogenetic vertical deep migration to delay their development to adulthood [31]. Diapause is a unique physiological process characterized by persistently reduced metabolism, increased stress resistance, and arrested development at a specific life stage [32]. Prior to entering diapause, lipids are sequestered in the form of wax esters in an oil sac [33] and constitute an important energy source. In the transcriptome of* C. sinicus*, several genes involved in fatty acid metabolism were identified by KEGG analysis. They are essential for lipid synthesis, transport, and storage, which are key components of preparation for diapause. Tarrant et al. [34] detected more highly expressed elongation of very long-chain fatty acids (ELOV) and fatty acid binding protein (FABP) genes in the bodies of active* C. finmarchicus* than those of diapausing individuals. ELOV is a member of a family of enzymes involved in the regulation of fatty acid elongation in both animals and plants [35]. Since some form of elongase is necessary for the synthesis of storage lipids, ELOV enzymes probably function in the synthesis of wax esters before entering diapause in* C. sinicus*. FABP belongs to a family of carrier proteins for fatty acids and other lipophilic substances such as retinoids [36]. These proteins are involved in the transfer of fatty acids between extra- and intracellular membranes. In* C. sinicus*, FABPs may function in facilitating the transport of wax esters to oil sac and the transport of lipophilic hormones, such as retinoids. Future studies are needed to identify the full complement of lipid metabolism genes in* C. sinicus* and particularly the mechanisms that regulate diapause.

Transcriptome sequencing provides an important resource for rapid and cost-effective development of molecular markers. The application of molecular markers will aid in clarifying the population genetic diversity, evaluating the genetic differentiation among geographical populations, and elucidating the impact of environmental elements on the genetic structure and geographical differentiation in marine ecology studies [37]. Pelagic marine organisms are expected to have great potential for gene flow owing to lack of physical barriers for genetic exchange in the “open” oceans. The planktonic copepod* C. sinicus* is the main contributor to zooplankton biomass in the shelf ecosystem of the Northwest Pacific Ocean and has a wide range of distribution, large population size, and prolific fecundity. It shows great geographical diversity in many biological and ecological phenotypes, such as the number of generations, timing of reproduction, vertical distribution, seasonal patterns of abundance, and other life history traits [38–40]. Recently, our marine biodiversity monitoring program revealed that the temporal and spatial distributions of* C. sinicus* varied obviously in the China Seas, which may be a consequence of global climate warming since this species is a warm-temperate one. Insights into the dispersal capabilities gained from population genetic studies will be crucial in predicting the response of* C. sinicus* populations to future climate change.

Although high-throughput SNP genotyping systems have become available with the development of large-scale sequencing technology [41, 42], these systems remain cost intensive. In this study, we found that *T*_*m*_-shift analysis is an efficient, cost-effective, and reliable method for SNP validation, especially for projects focused on a limited number of loci. The *T*_*m*_-shift method involves a single allele-specific PCR reaction followed by melting curve analysis. Wang et al. [21] indicated that up to 10,000 samples can be genotyped per day using a single 384-well real-time thermal cycler at a high accuracy (>99.9%). In the present study, 16 primer pairs could amplify target products and showed biallelic polymorphisms out of 51 primer pairs tested. The amplification failed in most cases (56.9%, 29/51) due to their monomorphisms, suggesting that the next generation sequencing (NGS) data could suffer from high SNP detection error rates by base-calling and alignment errors [43]. The uneven height of melting peaks in some primer pairs could make genotype scoring difficult when one primer amplified substantially more efficiently than the other. This issue was resolved by adding the more efficient primer at half of its original concentration (0.1 *μ*M). This is not always necessary as the genotypes can be identified even under the original conditions.

## Supplementary Material

Gene ontology (GO) enrichment analysis was done on Biological_process, Cellular_component and Molecular_function levels. GO terms involved in response to stimulus were identified. KEGG pathway analysis was also carried out in addition to GO analysis for the annotated unigenes. Genes involved in the lipid metabolism, immune system and development were identified.

## Figures and Tables

**Table 1:** Summary of RNA-Seq of the copepod *Calanus  sinicus* transcriptome.

Category	Number/length
Reads from raw data	58,944,478
Average read length (bp)	100
Reads after trimming	57,773,604
Percentage retained	98.0%
Average read length after trimming (bp)	97.9
Contigs after removing redundancy	69,751
Average length (bp)	928.8
Final N50 (bp)	1,127
Unigenes	43,417

**Table 2:** Representative transcripts involved in stress response and regulation of diapause in the *Calanus  sinicus* transcriptome.

Gene function	Number of unigenes	Size range (bp)
Response to stimulus		
Heat shock protein 90	10	92–714
Heat shock protein 70	17	120–900
Heat shock protein 60	1	584
Heat shock protein 40	1	410
Heat shock protein 10	1	112
Cytochrome P450 (CYP)	71	103–551
Glutathione S-transferase (GST)	31	103–409
Ferritin	14	105–226
Copper/zinc superoxide dismutase (Cu/Zn-SOD)	12	156–280
Mitochondrial manganese superoxide dismutase (Mn-SOD)	1	230
Catalase	4	207–696
Diapause/lipid metabolism		
Long-chain-fatty-acid-Coa ligase 3-like	35	115–726
Fatty acid binding protein (FABP)	3	86–135
Long-chain fatty acid transport protein 4-like	8	167–659
Elongation of very long chain fatty acids protein (ELOV)	19	88–363
Short-chain dehydrogenase/reductase family 16C member 6-like	8	116–312
Xanthine dehydrogenase (XAD)	12	139–1318
Hippocalcin	1	118
Ecdysteroid receptor (Ecr)	3	277–890

**Table 3:** KEGG biochemical mapping for *Calanus  sinicus*.

KEGG pathways	Subpathways	Number of isogenes	Number of genes
Metabolism	Metabolism of cofactors and vitamins	283	191
	Amino acid metabolism	764	492
	Nucleotide metabolism	424	282
	Metabolism of terpenoids and polyketides	134	87
	Glycan biosynthesis and metabolism	401	260
	Lipid metabolism	740	513
	Xenobiotics biodegradation and metabolism	285	199
	Energy metabolism	541	373
	Carbohydrate metabolism	901	559
	Metabolism of other amino acids	365	207
	Biosynthesis of other secondary metabolites	132	102
	Overview	270	161

Genetic information processing	Replication and repair	327	194
	Translation	1024	679
	Transcription	512	341
	Folding, sorting, and degradation	1051	686

Environmental information processing	Signal transduction	1534	979
	Signaling molecules and interaction	247	201
	Membrane transport	90	67

Cellular processes	Cell growth and death	702	444
	Cell motility	282	170
	Transport and catabolism	1121	736
	Cell communication	774	478

Organismal systems	Nervous system	774	505
	Excretory system	312	194
	Sensory system	198	136
	Digestive system	672	470
	Circulatory system	380	259
	Endocrine system	953	625
	Immune system	698	437
	Development	451	298
	Environmental adaptation	331	198

**Table 4:** Summary of simple sequence repeat (SSR) types in the *Calanus  sinicus* transcriptome.

SSR type	Number of SSRs	Percentage of total SSRs (%)
Dinucleotide	236	4.8
Trinucleotide	4,500	92.4
Tetranucleotide	118	2.4
Pentanucleotide and hexanucleotides	17	0.3

Total	4,871	

**Table 5:** Single nucleotide polymorphism (SNP) markers derived from the transcriptome of *Calanus  sinicus*.

Locus	Putative function	SNP type	Primer sequence (5′-3′)	*H*_*o*_	*H*_*e*_	Minor allele and frequency
CsSNP01	Heat shock protein 40	G/A	AS1: GCGGGCAGGGCGGCATGATGGAATGGACGGAACGGAG AS2: GCGGGCATGATGGAATGGACGGAATGTAA CR: CCTAAGCTGGCAAATGGATCATC	0.156	1.257	A 0.423
CsSNP02	Cytosolic heat shock protein 90 kda	G/T	AS1: GCGGGCAGGGCGGCCCTGATCTTGTCCAGAGCAGCG AS2: GCGGGCCCTGATCTTGTCCAGAGCATAT CR: GAAATATTTCTGAGAGAACTCATC	0.333	0.282	G 0.250
CsSNP03	Heat shock protein 70	C/T	AS1: GCGGGCAGGGCGGCTCCATCCTCACTATTGAAGACGGC AS2: GCGGGCTCCATCCTCACTATTGAAGATAGT CR: CAAGATGAGTGTCTCCACTGGTGG	0.500	0.523	T 0.452
CsSNP04	Ferritin	G/T	AS1: GCGGGCAGGGCGGCCCACTCAGCCAGCTCATCACGG AS2: GCGGGCCCACTCAGCCAGCTCATCATAT CR: GGCTCATCAACACCTTCAACAAC	0.194	0.325	G 0.338
CsSNP05	10 kda heat shock protein	C/T	AS1: GCGGGCAGGGCGGCGGATGAAGGATCCACTCTCCGCC AS2: GCGGGCGGATGAAGGATCCACTCTCTACT CR: GAGTCGTGGTGGCTGTTGGACC	0.822	0.522	T 0.375
CsSNP06	Prophenoloxidase	G/A	AS1: GCGGGCAGGGCGGCCAGTACCCCATCTTTACTCTGCTG AS2: GCGGGCCAGTACCCCATCTTTACTCTATTA CR: ATTAGCTATTGACCAAATGGAAATC	0.098	0.475	A 0.163
CsSNP07	Selenium- dependent salivary glutathione peroxidase	G/A	AS1: GCGGGCAGGGCGGCCCGTACTGGCCAGCCATACCGG AS2: GCGGGCCCGTACTGGCCAGCCATATAGA CR: ATCAGCATAGGTTCAACAGAAGAG	0.125	0.225	G 0.221
CsSNP08	Selenium-dependent glutathione peroxidase	G/A	AS1: GCGGGCAGGGCGGCTCGGCCTGGAAATGGGTGCGAG AS2: GCGGGCTCGGCCTGGAAATGGGTTAGAA CR: TTTGCTTCCACCTTATCGAACATC	0.246	0.325	A 0.223
CsSNP09	Superoxide dismutase	G/A	AS1: GCGGGCAGGGCGGCAAAGTCAGAGGAACCATTACGCTG AS2: GCGGGCAAAGTCAGAGGAACCATTACAATA CR: TCCTTCCAATAACACGGACGTGCC	0.056	0.468	A 0.200
CsSNP10	Ferritin heavy subunit	C/T	AS1: GCGGGCAGGGCGGCCATTTCTTGGAGGAACAGGTAGGC AS2: GCGGGCCATTTCTTGGAGGAACAGGTATAT CR: CATCTCCACTCTGGTAAGCAAGTCC	0.083	0.475	C 0.324
CsSNP11	Lysosomal aspartic protease precursor	C/T	AS1: GCGGGCAGGGCGGCGATGGACCCACGCTATGACCGGC AS2: GCGGGCGATGGACCCACGCTATGACCAGT CR: GCATCCATATAATTAGACAGTGG	0.500	0.482	T 0.265
CsSNP12	Catalase	C/T	AS1: GCGGGCAGGGCGGCTATGAATGGATATGGATCCCGC AS2: GCGGGCTATGAATGGATATGGATCACAT CR: GATGGGCTCATTGTCTGCATTC	0.250	0.335	C 0.188
CsSNP13	Trypsin	C/T	AS1: GCGGGCAGGGCGGCCTGCACGAAGCAATGCTGTCGAC AS2: GCGGGCCTGCACGAAGCAATGCTGTTAAT CR: AGGGGTCTTTGTAGTTTGGATCTC	0.833	0.501	T 0.415
CsSNP14	Zwilch-like protein	C/T	AS1: GCGGGCAGGGCGGCTGGAGGCTTTTAGCCTGGAGGCC AS2: GCGGGCTGGAGGCTTTTAGCCTGGATACT CR: GACCACAGCATGTCCGTGAAGTCC	0.778	0.516	C 0.425
CsSNP15	Broad-complex core protein isoform 6	C/T	AS1: GCGGGCAGGGCGGCTATACCTTCCTCCAGAGGCCGCC AS2: GCGGGCTATACCTTCCTCCAGAGGCTACT CR: GTCAGTGTATTTCACACCCTTGAG	0.223	0.212	T 0.325
CsSNP16	V-Type proton Atpase 116 kda subunit	C/T	AS1: GCGGGCAGGGCGGCTATACCTTCCTCCAGAGGCCGCC AS2: GCGGGCTATACCTTCCTCCAGAGGCTACT CR: GTCAGTGTATTTCACACCCTTGAG	0.456	0.487	C 0.212

GC tails are underlined, and additional deliberate mismatches are boxed. AS1 and AS2: allele-specific primers; CR: common reverse primer; *H*_*o*_: observed heterozygosity; *H*_*e*_: expected heterozygosity.

## References

[1] Humes AG (1994). How many copepods?. *Hydrobiologia*.

[2] Bron JE, Frisch D, Goetze E, Johnson SC, Lee CE, Wyngaard GA (2011). Observing copepods through a genomic lens. *Frontiers in Zoology*.

[3] Yasuike M, Leong J, Jantzen SG (2012). Genomic resources for sea lice: analysis of ESTs and mitochondrial genomes. *Marine Biotechnology*.

[4] Carpio Y, Basabe L, Acosta J (2011). Novel gene isolated from *Caligus rogercresseyi*: a promising target for vaccine development against sea lice. *Vaccine*.

[5] Hansen BH, Altin D, Nordtug T, Olsen AJ (2007). Suppression subtractive hybridization library prepared from the copepod *Calanus finmarchicus* exposed to a sublethal mixture of environmental stressors. *Comparative Biochemistry and Physiology D: Genomics and Proteomics*.

[6] Lenz PH, Unal E, Hassett RP (2012). Functional genomics resources for the North Atlantic copepod, *Calanus finmarchicus*: EST database and physiological microarray. *Comparative Biochemistry and Physiology D: Genomics and Proteomics*.

[7] Lenz PH, Roncalli V, Hassett (2014). *De novo* assembly of a transcriptome for *Calanus finmarchicus* (Crustacea, Copepoda)—the dominant zooplankter of the North Atlantic Ocean. *PLoS ONE*.

[8] Unal E, Bucklin A, Lenz PH, Towle DW (2013). Gene expression of the marine copepod *Calanus finmarchicus*: responses to small-scale environmental variation in the Gulf of Maine (NW Atlantic Ocean). *Journal of Experimental Marine Biology and Ecology*.

[9] Christie AE, Roncalli V, Wu LS, Ganote CL, Doak T, Lenz PH (2013). Peptidergic signaling in *Calanus finmarchicus* (Crustacea, Copepoda): *in silico* identification of putative peptide hormones and their receptors using a de novo assembled transcriptome. *General and Comparative Endocrinology*.

[10] Christie AE, Roncalli V, Lona PB (2013). *In silico* characterization of the insect diapause-associated protein couch potato (CPO) in *Calanus finmarchicus* (Crustacea: Copepoda). *Comparative Biochemistry and Physiology D: Genomics and Proteomics*.

[11] Christie AE, Fontanilla TM, Roncalli V, Cieslak MC, Lenz PH (2014). Identification and developmental expression of the enzymes responsible for dopamine, histamine, octopamine and serotonin biosynthesis in the copepod crustacean *Calanus finmarchicus*. *General and Comparative Endocrinology*.

[12] Ning J, Wang MX, Li CL, Sun S (2013). Transcriptome sequencing and de novo analysis of the copepod *Calanus sinicus* using 454 GS FLX. *PLoS ONE*.

[13] Uye S (2000). Why does *Calanus sinicus* prosper in the shelf ecosystem of the Northwest Pacific Ocean?. *ICES Journal of Marine Science*.

[14] Xu Z, Ma Z, Wu Y (2011). Peaked abundance of *Calanus sinicus* earlier shifted in the Changjiang River (Yangtze River) Estuary: a comparable study between 1959, 2002 and 2005. *Acta Oceanologica Sinica*.

[15] Yang Q, Wang ZL, Fan JF, Shao KS, Li HJ (2012). Zooplankton diversity and its variation in the Northern Yellow Sea in the autumn and winter of 1959, 1982 and 2009. *Acta Ecologica Sinica*.

[16] Ekblom R, Galindo J (2011). Applications of next generation sequencing in molecular ecology of non-model organisms. *Heredity*.

[17] Gao XG, Han JB, Lu ZC, Li YF, He CB (2013). De novo assembly and characterization of spotted seal *Phoca largha* transcriptome using Illumina paired-end sequencing. *Comparative Biochemistry and Physiology D: Genomics and Proteomics*.

[18] Grabherr MG, Haas BJ, Yassour M (2011). Full-length transcriptome assembly from RNA-Seq data without a reference genome. *Nature Biotechnology*.

[19] Conesa A, Götz S, García-Gómez JM, Terol J, Talón M, Robles M (2005). Blast2GO: a universal tool for annotation, visualization and analysis in functional genomics research. *Bioinformatics*.

[20] Faircloth BC (2008). Msatcommander: detection of microsatellite repeat arrays and automated, locus-specific primer design. *Molecular Ecology Resources*.

[21] Wang J, Chuang K, Ahluwalia M (2005). High-throughput SNP genotyping by single-tube PCR with T_m_-shift primers. *BioTechniques*.

[22] Yeh FC, Yang RC, Boyle T (1999). *PopGene Version 131: Microsoft Window-Based Freeware for Population Genetic Analysis*.

[23] Hwang J, Wong CK (2005). The China coastal current as a driving force for transporting *Calanus sinicus* (Copepoda: Calanoida) from its population centers to waters off Taiwan and Hong Kong during the winter northeast monsoon period. *Journal of Plankton Research*.

[24] Yin J, Huang L, Li K, Lian S, Li C, Lin Q (2011). Abundance distribution and seasonal variations of *Calanus sinicus* (Copepoda: Calanoida) in the northwest continental shelf of South China Sea. *Continental Shelf Research*.

[25] Wiacek M, Uddin N, Kim HJ, Zubrzycki IZ (2013). Proteome changes in response to ecologically viable environmental variation in *Calanus sinicus*. *Protein and Peptide Letters*.

[26] Pyza E, Mak P, Kramarz P, Laskowski R (1997). Heat shock proteins (HSP70) as biomarkers in ecotoxicological studies. *Ecotoxicology and Environmental Safety*.

[27] Li HJ, Liu SX, He CB, Gao XG, Yuan XT (2013). Identification of a small HSP gene from hard clam *Meretrix meretrix* and its potential as an environmental stress biomarker. *Aquatic Biology*.

[28] Gonzalez FJ (2005). Role of cytochromes P450 in chemical toxicity and oxidative stress: studies with CYP2E1. *Mutation Research—Fundamental and Molecular Mechanisms of Mutagenesis*.

[29] Park SY, Nair PMG, Choi J (2012). Characterization and expression of superoxide dismutase genes in *Chironomus riparius* (Diptera, Chironomidae) larvae as a potential biomarker of ecotoxicity. *Comparative Biochemistry and Physiology D: Genomics and Proteomics*.

[30] Kim B, Rhee J, Park GS, Lee J, Lee Y, Lee J (2011). Cu/Zn- and Mn-superoxide dismutase (*SOD*) from the copepod *Tigriopus japonicus*: molecular cloning and expression in response to environmental pollutants. *Chemosphere*.

[31] Hirche HJ (1996). Diapause in the marine copepod, *Calanus finmarchicus*: a review. *Ophelia*.

[32] Aruda AM, Baumgartner MF, Reitzel AM, Tarrant AM (2011). Heat shock protein expression during stress and diapause in the marine copepod *Calanus finmarchicus*. *Journal of Insect Physiology*.

[33] Miller CB, Morgan CA, Prahl FG, Sparrow MA (1998). Storage lipids of the copepod *Calanus finmarchicus* from Georges Bank and the Gulf of Maine. *Limnology and Oceanography*.

[34] Tarrant AM, Baumgartner MF, Verslycke T, Johnson CL (2008). Differential gene expression in diapausing and active *Calanus finmarchicus* (Copepoda). *Marine Ecology Progress Series*.

[35] Leonard AE, Pereira SL, Sprecher H, Huang Y (2004). Elongation of long-chain fatty acids. *Progress in Lipid Research*.

[36] Chmurzyńska A (2006). The multigene family of fatty acid-binding proteins (FABPs): function, structure and polymorphism. *Journal of Applied Genetics*.

[37] Zhan A, Hu J, Hu X (2009). Fine-scale population genetic structure of zhikong scallop (chlamys farreri): do local marine currents drive geographical differentiation?. *Marine Biotechnology*.

[38] Huang C, Uye S, Onbe T (1993). Ontogenetic diel vertical migration of the planktonic copepod *Calanus sinicus* in the inland sea of Japan. 3. Early summer and overall seasonal pattern. *Marine Biology*.

[39] Zhang G, Sun S, Zhang F (2005). Seasonal variation of reproduction rates and body size of *Calanus sinicus* in the Southern Yellow Sea, China. *Journal of Plankton Research*.

[40] Huo Y, Wang S, Sun S, Li C, Liu M (2008). Feeding and egg production of the planktonic copepod *Calanus sinicus* in spring and autumn in the Yellow Sea, China. *Journal of Plankton Research*.

[41] Martino A, Mancuso T, Rossi AM (2010). Application of high-resolution melting to large-scale, high-throughput SNP genotyping: a comparison with the TaqMan method. *Journal of Biomolecular Screening*.

[42] Davey JW, Hohenlohe PA, Etter PD, Boone JQ, Catchen JM, Blaxter ML (2011). Genome-wide genetic marker discovery and genotyping using next-generation sequencing. *Nature Reviews Genetics*.

[43] Nielsen R, Paul JS, Albrechtsen A, Song YS (2011). Genotype and SNP calling from next-generation sequencing data. *Nature Reviews Genetics*.

